# A Retrospective Study of Suspected Animal Abuse Cases in Trinidad and Tobago, 2008–2025 †

**DOI:** 10.3390/ani16071088

**Published:** 2026-04-02

**Authors:** Karelma Frontera-Acevedo, Alanis Boodram, Xaria Luke, Emily Phillip, Rod Suepaul, Lana Gyan

**Affiliations:** 1 Department of Basic Veterinary Sciences, School of Veterinary Medicine, Faculty of Medical Sciences, University of the West Indies, Building 47, Eric Williams Medical Sciences Complex, Uriah Butler Highway, St. Augustine 310140, Trinidad and Tobago; 2 Veterinary Diagnostic Laboratory, Ministry of Agriculture and Fisheries, Building 49, Eric Williams Medical Sciences Complex, Uriah Butler Highway, St. Augustine 310140, Trinidad and Tobago

**Keywords:** Caribbean, veterinary forensic pathology, animal abuse, non-accidental injury

## Abstract

Forensic veterinary pathology is becoming increasingly important in investigating situations of suspected animal abuse. In Trinidad and Tobago, there are only two veterinary diagnostic laboratories capable of performing necropsies, one at the University of the West Indies School of Veterinary Medicine (UWI-SVM) and the other at the Ministry of Agriculture and Fisheries, Veterinary Diagnostic Laboratory (MAF-VDL). The databases of both UWI-SVM and MAF-VDL were searched for cases of suspected animal abuse between 2008 and 2025. Based on pathology records and case submission information, cases were categorized by year, species, cause of death (COD) or manner of injury (MOI), police submissions, and types of trauma and poisonings. A total of 113 cases of suspected animal abuse were submitted in the years 2008–2025. Case submissions reached a peak in 2015 (*n* = 23). The most submitted animal species was dogs (77%). Of the 113 cases, only 24 (20%) had a confirmed COD/MOI. The most common confirmed COD/MOI was trauma. The few confirmed cases of poisoning (*n* = 6) include carbamates, warfarin, malathion, and ethylene glycol. Owners appear to be aware of the importance of pathology to help diagnose suspected animal abuse cases. One major limitation in advancing forensic veterinary pathology on the island, and a factor preventing the prosecution of suspected animal abuse cases, is the lack of a toxicological laboratory capable of handling animal samples.

## Simple Summary

Forensic veterinary pathology is becoming increasingly important in investigating situations of suspected animal abuse throughout the world, including in the Republic of Trinidad and Tobago. However, locally, there is a lack of submitted cases and, therefore, published data involving suspected animal abuse and the type of abuse observed. This paper reviews suspected animal abuse cases that were submitted to the necropsy services in Trinidad and Tobago between 2008 and 2025.

## 1. Introduction

Animal abuse can reflect both societal concerns and individual well-being, and should be investigated and prosecuted because the welfare of animals is an important component of public responsibility and humane treatment; it can also be related to other interpersonal crimes of violence [1,2,3,4,5]. Animal abuse reflects a culture of violence and abuse, and is carried out by people who do not expect authorities to intervene and those who have the intention of intimidating the owners or companions of the animals being abused [2,4]. The investigation of animal cruelty is often based on the concept of the five freedoms [6]. Based on this concept, countries around the world have enacted laws that protect animals and cover these concepts, to a greater or lesser extent [2,4,5,7].

In the Republic of Trinidad and Tobago, according to the initial Summary Offences Act of 1921, Chapter 11:02, Sections 79–90 inclusive [8], it is illegal to kill, maim, starve, ill-treat, abuse, torture, or wound any dog, bird, or other animal that is being kept for any domestic purpose. The punishments for the various offenses included fines, which did not exceed TTD $500 (slightly less than USD $75), or up to two months of jail [8]. This was later updated and expanded in the Animal (Diseases, Importation, Health, and Welfare) Act [9], which now also includes beating, neglect, and overloading. This act also includes stronger penalties, with up to five years in jail and a TTD fine of $200,000 (slightly less than USD $30,000). These changes were meant to demonstrate the government’s recognition that animal suffering is a matter of public concern.

Forensic veterinary pathology is becoming increasingly important in recognizing and investigating animal abuse cases [5,10,11,12]. Sometimes these cases are conducted following police reports because of suspicious criminal behavior [7], but they can also be requested by the owner, as a means of obtaining closure, particularly if the circumstances surrounding the pet’s death are suspicious [13]. In Trinidad and Tobago, there is no restriction as to who can submit for a forensic necropsy; however, cases with legal implications are referred through the police. Forensic necropsies are similar to regular diagnostic necropsies, but they may require ancillary testing that may be unusual, such as diagnostic imaging, toxicological studies, or even DNA sequencing. This testing can be costly, and the owner would typically bear the costs [5,10,11,13]. This can potentially be a limitation in the submission of suspected cases.

To the authors’ knowledge, this is the first published article regarding forensic veterinary pathology in the Republic of Trinidad and Tobago. This article is a revised and expanded version of a poster entitled “A Review Of The Cause Of Death And Mode Of Injury Of Suspected Animal Abuse Cases In Trinidad And Tobago, 2008–2015”, which was presented at the 67th ACVP Annual Meeting, New Orleans, LA, United States, in December 2016 [14]. Suspected animal abuse cases submitted to the University of the West Indies School of Veterinary Medicine (UWI-SVM) and the Ministry of Agriculture and Fisheries Veterinary Diagnostic Laboratory (MAF-VDL) during 2008–2025 were identified and categorized according to predefined criteria. The main aim of this article is to raise awareness of the suspected cases of animal abuse observed in the country, as well as the limitations present. This could lead to further discussion on how to improve diagnostic practices and the prosecution of individuals who violate animal welfare laws [8,9].

## 2. Materials and Methods

### 2.1. Sample Population and Case Selection

This is a retrospective study based on necropsy reports from the databases of UWI-SVM and MAF-VDL for potential suspected animal abuse cases in Trinidad and Tobago during the period 2008 to 2025. Included cases were submitted by owners, non-governmental organizations (NGOs) associated with animal rescue or welfare, or law enforcement agents who suspected animal abuse as defined by national legislation [8,9]. The databases of both diagnostic laboratories are organized and stored differently. For the UWI-SVM database, the standardized keywords used included “toxins”, “toxic”, “toxicology”, “poisons”, “poisoning”, “trauma”, “blunt force trauma”, “traumatic”, and “gunshot”. For the MAF-VDL, during part of the study period, the server was not accessible online, but they did have a chain of custody notebook where they logged, identified, and detailed all animal abuse cases submitted by the police. In addition, they had a necropsy logbook, and necropsies during the study period were manually reviewed to determine if they involved cases of animal abuse, neglect, poisoning, gunshot wounds, or trauma. The initial necropsy reports from both databases were then reviewed to check if they fit the inclusion and exclusion criteria.

The inclusion criteria were the following:

- Cases submitted between 2008 and 2025.
- Cases involving the following domestic animal species: dogs, cats, horses, cows, sheep, goats, pigs, and pet birds.
- Cases had to meet at least one of the following criteria:
  ○ Police cases, where there was a police report made, and the police participated in the process;
  ○ The submitted case history indicated animal abuse as a differential diagnosis for COD or MOI;
  ○ The veterinary pathologist, after performing the necropsy, suspected the COD or MOI was related to animal abuse.
- The cases included completed necropsy reports.

The exclusion criteria were as follows:

- Suspected cases with partial reports or without reports accompanying the case identification number;
- Biopsy or cytology of live animals;
- Cases that were suspected initially to be related to animal abuse, but after necropsy, an alternative cause of death unrelated to animal abuse was identified;
- Cases involving exotic animal species or environmentally sensitive species covered under other laws [15].

The remaining cases were categorized into cases per year; species; cause of death (COD) or manner of injury (MOI) if euthanized; confirmed or unconfirmed cases; and whether the cases were submitted by the police or private cases (owners or NGOs). For COD, they were divided into trauma and poisoning cases. Trauma included gunshot wounds, blunt force trauma (BFT), and strangulation. For the suspected poisoning cases, the accompanying history or police report had to strongly suggest poisoning, and necropsy reports stated poisoning as the most likely COD. Confirmed cases included cases for which there was a positive toxicological result (in the case of poisoning) or one where, after necropsy, the veterinary pathologist interpreted that the injuries observed caused the death of the animal. Unconfirmed cases of suspected animal abuse consisted exclusively of cases that were suspected of poisoning for which no toxicology was obtained, as trauma cases were able to be confirmed at necropsy. Police cases were those that were referred by the police because a report was made or the death involved some criminal activity (house invasion, for example) where the police were called. Private cases are those submitted by citizens, usually the owners of these animals, and sometimes NGOs that worked in the area where the animal was found, if the animal was a stray.

All cases submitted by police personnel are treated as veterinary forensic necropsies from the start. Privately submitted cases in which the person submitting the carcass suspects animal abuse are also treated as veterinary forensic necropsies. These necropsies were performed by board-certified or board-eligible veterinary pathologists, following guidelines from published forensic necropsy methodologies described in the literature [13,16]. Police officers were always present during police submitted cases, and they immediately collected bullet fragments. Toxicological samples were collected in clearly identified plastic bags. While the toxicological laboratory was working, the samples were sent immediately to that facility, but after the toxicological laboratory closed, the samples were stored in the refrigerators of the necropsy laboratories until the clients collected them.

### 2.2. Statistical Analysis

Descriptive statistical analysis was performed using Microsoft Excel for Microsoft 365 MSO (Version 2511 Build 16.0.19426.20218) 64-bit.

## 3. Results

### 3.1. Total Number of Cases Between 2008 and 2025 and Species

**Figure 1:**
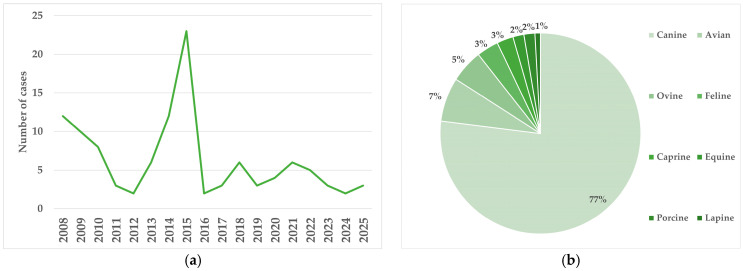
Number of necropsy cases of suspected animal abuse cases, broken down by years (**a**) and by species (**b**).

In total, 113 cases were included in this survey, as they met the previously established inclusion criteria. The breakdown of the cases per year and per species is presented in Figure 1. The year with the most cases submitted was 2015 (*n* = 23; 20.35%), while 2012, 2016, and 2024 were the years with the fewest cases submitted (*n* = 2; 1.77%). The average number of suspected animal abuse cases submitted per year was 6.28. The most common species was dogs (*n* = 87; 77%).

### 3.2. Confirmed Versus Unconfirmed Cases

**Figure 2:**
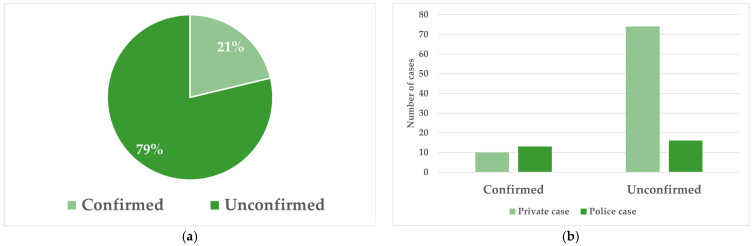
(**a**) The proportion of confirmed versus unconfirmed necropsy cases of suspected animal abuse. (**b**) The breakdown of suspected animal abuse cases, if they were submitted by the police or privately, and whether they were confirmed or not.

Out of the 113 suspected animal abuse necropsy cases, only 24 cases (21%) had a confirmed COD/MOI that indicated it was likely an animal abuse case (Figure 2). Out of the 113 cases included, 29 (26%) were submitted as police cases, while 84 (74%) cases were submitted as private cases. As shown in Figure 2, when the cases were confirmed, the difference between police and private cases was small, as was the difference between police cases that were confirmed or not. However, most unconfirmed cases were private submissions, usually submitted by the owners (*n* = 74; 82%).

### 3.3. Cause of Death/Manner of Injury

**Figure 3:**
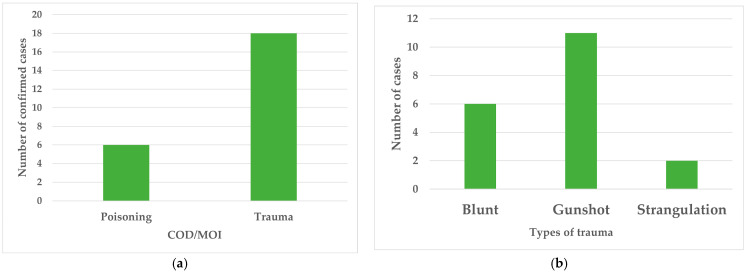
(**a**) Breakdown of the confirmed cases of animal abuse necropsies. (**b**) The distinct types of trauma identified in the confirmed animal abuse cases.

The two main broad categories of COD/MOI found were poisoning and trauma. Of the 113 cases, 1 case had signs of both trauma and possible poisoning. All unconfirmed cases (*n* = 89) involved suspected poisoning. Most of the 24 confirmed cases were associated with trauma (*n* = 18; 75%). The types of traumas observed included BFT, gunshot, and strangulation, with the most common being gunshot wounds (*n* = 11; 61%, Figure 3). Gross lesions of BFT included a fractured skull, other fractures, hemoabdomen, and hemothorax.

Radiographs were taken, if an X-ray machine was available, to detect all or most of the bullet fragments in suspected gunshot wounds, and these were collected and given to the police officer present. Lesions present in gunshot wounds included multiple comminuted fractures and severe organ(s) rupture and hemorrhages along the bullet pathway. If the bullets severed major vessels, hemoabdomen or hemothorax would be present. All the gunshot cases were dogs, and of the 11 gunshot cases, 9 of them were submitted by the police.

The two cases of death by strangulation involved hanging. The gross lesions found with hanging included circumferential ligature marks in the neck, with hemorrhage in adjacent musculature, and hemorrhage and edema found in the trachea, muscles, and subcutaneous tissues cranial to the ligature marks.

**Figure 4:**
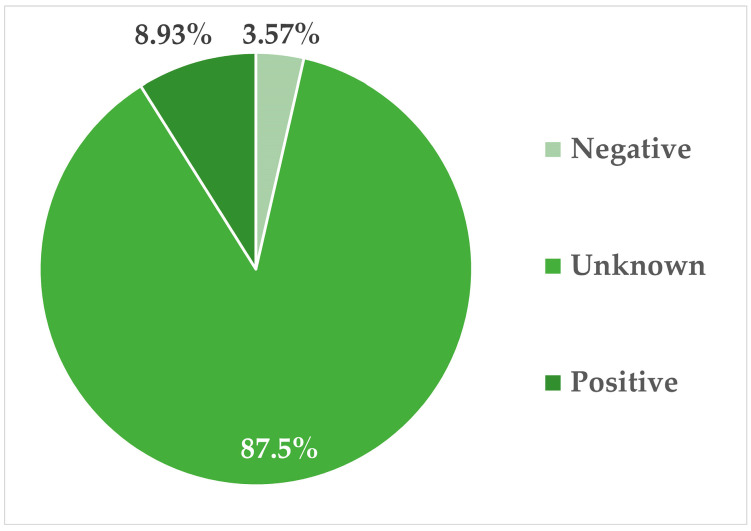
Graph showing the breakdown of toxicological results for suspected cases of poisoning with samples collected for toxicology.

There were 95 cases of suspected poisoning presented between 2008 and 2025. The suspicion was primarily based on the history provided by the submitter, along with the findings of necropsy. Some of these necropsy findings included severe massive hemorrhages throughout the body, like multiple hematomas, pulmonary hemorrhages, hyphema, hemoabdomen, and hemothorax. Another common finding was marked generalized hyperemia. Only 56 (59%) had samples collected for toxicology. Samples collected for toxicology included stomach contents (if present), the liver, and the kidneys. Of the cases submitted, only seven (12.5%) had results, five of which were positive for a toxin. There is no record of toxicological results for the rest of the samples (*n* = 49; 87.5%), as shown in Figure 4. The toxins identified in the toxicological samples were carbamate (*n* = 3), malathion (*n* = 1), and warfarin (*n* = 1). One of the poisoning cases was confirmed to be ethylene glycol toxicity based on histopathology, bringing the total confirmed poisoned cases to six, as shown in Figure 3.

## 4. Discussion

The number of suspected animal abuse cases submitted to the combined veterinary diagnostic laboratories in Trinidad and Tobago has fluctuated over the study period (Figure 1). This could be due to a combination of factors, some of which cannot be directly assessed or which could be identified in another study. In terms of facilities and personnel, MAF-VDL and UWI-SVM are the only two veterinary pathology laboratories in the country providing necropsy services. Around 2015, there were three veterinary anatomical pathologists practicing on the island (two of them board-certified and one board-eligible), and there was also a functioning toxicology laboratory that processed veterinary toxicological samples. This may have contributed, along with greater media awareness, to the submission of more cases of necropsy during that year.

The reasons for decreased submissions can also be varied. During the period 2011–2012, there was only one pathologist working in the country at the UWI-SVM. At the same time, there was no pathologist at the MAF-VDL, so it is likely that cases were turned away. Another potential reason for a decrease in submissions of suspected animal abuse cases is the closure of the only available veterinary toxicology laboratory (at UWI-SVM) in 2016. This issue is discussed further in the subsection below.

Only 29 of the 113 cases (25.66%) were submitted by police officers. At least one police officer was present at every necropsy submitted by them. If bullet fragments were found at necropsy, the police collected them immediately at the end of the necropsy. The veterinary pathologists did not have the expertise to identify the weapons based on the fragments, nor did they have access to the ballistics assay that the police would have done to identify the weapons.

Most of the cases (*n* = 84) were submitted by private citizens, usually the owners, but occasionally also rescue organizations active in the area where the animals died (in the case of stray animals). A necropsy at the owner’s request is not uncommon, and may be part of an effort from the owner to find closure after their pet’s death [13]. This is why, particularly in privately submitted cases, instances where another COD was found were not included in the study, even if initially the owner expected it to be related to animal abuse. The owners may also have previously suspected animal abuse as part of their pet’s demise [13] and wanted to confirm it in order to submit a police report.

Despite lacking toxicological confirmation, by far the most common suspected cause of death by animal abuse during the years studied was poisoning. This type of animal abuse has not been as clearly linked to interpersonal violence as other types of death, but the motivations may be similar, such as revenge against the animal or owner or nonspecific sadism [1]. The toxins that were identified are all pesticides or rodenticides, with carbamate being the most identified (*n* = 3), which is consistent with what has been reported in other studies [17,18,19]. Carbamate [20], malathion [21], and warfarin [22] are registered in the Republic of Trinidad and Tobago for use in agriculture or domestic areas as pesticides and rodenticides. In the context of Trinidad and Tobago, these chemicals are easier to obtain than in other countries and would not be difficult for civilians to obtain and use to commit crimes. For example, carbamates are currently banned in numerous countries, and although still present in intentional poisonings (due to stockpiles), their percentage as a total has decreased [18,19].

Although unconfirmed because of a lack of toxicological testing, another chemical that was frequently suspected to cause death because of the gross lesions (severe pulmonary hemorrhage and emphysema) involved was paraquat dichloride (gramoxone). Following the publication of articles associating the ingestion of gramoxone with a high number of suicides in Trinidad [23], the chemical is now de-registered, and there is no permit to legally import the chemical to the island. However, during most of the timeline studied, gramoxone was available and was not as regulated as it is in other countries, making it accessible to people who wanted to commit crimes.

Most of the confirmed cases of suspected animal abuse involved some type of trauma (Figure 3a). The most common form of trauma was gunshot wounds, with 11 cases (Figure 3b, 57.89%). Out of the total 113 cases included in the study, gunshot wounds account for 9.7% of the total cases. Considering only the confirmed cases (*n* = 24), the gunshot cases represent almost half of the confirmed cases of animal abuse necropsy (45.83%). As mentioned before, most of the gunshot cases were submitted by the police, and they collected the bullet fragments recovered. The veterinary pathologists would then have no knowledge of what type of weapon was used. Although the use of postmortem radiographs is recommended for gunshot wounds [1,24], this was not always possible because the radiograph machine that was available was not working. For cases in which it was used, it aided the identification and collection of all the fragments. The use of firearms involves premeditation and is highly unlikely to be accidental [1], increasing the likelihood that such behavior can also be enacted in humans. Notably, cases submitted by the police involved animals in a police case, such as robbery or home invasion.

Blunt force trauma (BFT) was confirmed as the COD in six cases. There are different types of BFT, with the three main patterns being abrasions, contusions, and lacerations [25]. Unfortunately, the information gathered from the databases of UWI-SVM and MAF-VDL do not provide sufficient information to distinguish which of the types of BFT are most common. BFT is commonly associated with domestic abuse and child abuse, and it is an indicator of high risk for future violence against others [1]. Four out of the six cases with this type of lesion were police-submitted cases, again indicating that the animal died in a situation that led to police intervening, such as domestic violence, robbery, or home invasion.

Of the confirmed cases of trauma, two were the result of strangulation (Figure 3b). This is a type of asphyxiation that is caused by applying pressure to the neck in various ways [26]. The information provided by the database did not distinguish between the three types of strangulation (ligature, hanging, or manual), but a search on local newspapers revealed a news story about a public dog hanging that went viral, and according to the date, it corresponds to one of the strangulation cases [27]. All forms of asphyxia, including strangulation, require a high degree of premeditation and close contact, and are also considered to be high risk for future violence against humans and other animals [1].

Unlike other reports [28], no cases were found of suspected animal abuse by burning, either by thermal injuries or smoke inhalation. This could be because in the context where many of these suspected cases occur, poisoning or trauma are methods more easily available. Another possibility would be that in the cases of arson, people or property were likely to be directly affected, and the authorities may have opted to pursue criminal charges related to the damage caused to them and ignore the animals affected. Suspected animal abuse cases involving drowning were also absent in this study, again in contrast with other articles [17,28].

### Limitations of the Study

The main limitation in the current study, and in fact, the main limitation of further studies on veterinary forensics and animal abuse in Trinidad and Tobago, is the lack of an appropriate toxicological laboratory willing to assay veterinary samples. Without this important ancillary tool, it is not possible to determine whether suspected poisoning cases are truly poisoning cases, and which poisons are the most commonly used. The lack of toxicological results also limits the prosecution of the suspected cases because there is no definitive proof of a poison being used to kill the animals. Notably, even when a toxicological laboratory was functioning, this laboratory did not always assay the samples collected or provide the results in a timely manner. Of the 56 suspected poisoning cases with samples collected, only 7 have toxicological results (12.5%). Therefore, not only must a veterinary toxicological laboratory be present, but it must also be run efficiently.

The small number of confirmed cases of animal abuse necropsy compared to the number of unconfirmed suspected animal abuse necropsy cases, just 20%, is not unique to Trinidad. For example, in a study done in Portugal [17], less than a quarter of the cases were confirmed. The reasons given for the small number of confirmed cases are similar to those found in Trinidad, with a lack of appropriate toxicological analysis to confirm the large number of suspected poisoning cases.

## 5. Conclusions

The lack of toxicological data prevented definitive diagnosis of poisoning as a cause of death in the suspected cases of animal abuse submitted to veterinary pathologists in the Republic of Trinidad and Tobago. This highlights the need for a functioning toxicological laboratory in the country, accompanied by increased awareness and action from the police and legal system to prosecute the suspects.

Future research should involve identifying individuals who have been prosecuted and/or punished in Trinidad and Tobago under their animal welfare laws throughout the years, and the role of the veterinarians in those cases, including the use of necropsy reports during trial.

## Data Availability

The data presented in this study are available on request from the corresponding author due to privacy and legal reasons, as they can be part of police investigations.
